# Sleeping-Site Decisions in Tibetan Macaques: Social and Seasonal Drivers

**DOI:** 10.3390/ani16060899

**Published:** 2026-03-13

**Authors:** Huihui Chen, Tong Zhang, Peipei Yang, Xi Wang

**Affiliations:** 1 School of Resources and Environmental Engineering, Anhui University, No. 111, Jiulong Rd, Hefei 230601, China; 17760854940@163.com (H.C.); 18297815809@163.com (T.Z.); ahppyang@126.com (P.Y.); 2 International Collaborative Research Center for Huangshan Biodiversity and Tibetan Macaque Behavioral Ecology, Hefei 230601, China

**Keywords:** sleeping-site selection, decision-making, social network, seasonal variation, Tibetan macaque

## Abstract

Sleeping-site selection is a critical decision-making process in animals, influenced by evolutionary pressures. However, the key factors controlling this choice under group demography, and how these vary seasonally, remain poorly understood. This study investigated the selection of arboreal versus terrestrial sleeping sites and the underlying decision-making processes in a free-ranging group of Tibetan macaques (*Macaca thibetana*) at Huangshan, China, across mating (July–January) and non-mating (February–April) seasons. Generally, Tibetan macaques slept arboreally during mating season (July–September), switched to terrestrial sites afterward (October–March), and returned to trees in the following April. As for the decision-making, females consistently played a central role, initiating collective movements to arboreal sites more frequently and attracting more followers during the mating season, and joining the collective movements earlier across all seasons. Decision-making rules also varied seasonally. Females and middle-aged/older individuals mainly initiated movements to arboreal sites during the mating season, whereas terrestrial movements were led primarily by older individuals, with high social centrality attracting more followers. In the non-mating season, no specific social traits predicted initiators across sleeping sites, though females consistently attracted more followers and joined movements earlier. In terrestrial movements specifically, older individuals joined later, whereas highly socially central individuals joined earlier. Our research reveals that the sleeping-site selection decisions of Tibetan macaques are influenced by their matrilineal group structure. This study provides insights into the ecological adaptability of primates, demonstrating how dynamic decision-making supports survival in seasonal environments among social animals.

## Simple Summary

How do Tibetan macaques exhibit seasonal variations in sleeping-site selection and what are the underlying collective decision-making processes? In this study, we not only investigated the arboreal and terrestrial sleeping-site selection of free-ranging Tibetan macaques in Huangshan across mating and non-mating seasons, but also identified the key factors influencing these collective decisions. We found that these macaques switch their sleeping sites between the two seasons. Females played a central role throughout the study, initiating more arboreal movements in the mating season and joining movements earlier across all periods. Furthermore, older individuals and females dominated most terrestrial movements in the mating season; in the non-mating season, no specific social factors could predict movement initiators, yet females retained their advantage in attracting followers. These findings provide valuable insights into how social animals adjust collective decision-making to adapt to seasonal environments.

## 1. Introduction

Group living is a prevalent strategy among animals for enhancing fitness in response to extreme environmental conditions and resource scarcity [1]. To reach these benefits, however, groups must coordinate the timing and direction of their movements to achieve a collective consensus for unified actions [2]. Collective decision-making enables group members to respond effectively to environmental changes by maximizing individual and collective outcomes through consensus [3].

Sleeping-site selection in primates is shaped by multiple ecological and social factors, including predation risk, microclimate, and group cohesion [4,5]. It constitutes a critical daily collective decision for group-living animals and it serves as an essential nocturnal refuge for diurnal species [6,7]. Consequently, group coordination is indispensable to selecting a shared sleeping site for individual survival and energy conservation. Sleeping-site selection strategies vary significantly across primate species. For example, colobus monkeys (*Colobus vellerosus*) preferentially select tall and densely foliated trees to elevate their resting sites away from terrestrial predators, and passively delay their morning departure in colder conditions [8]. In contrast, Japanese macaques (*Macaca fuscata*) employ an active thermoregulatory strategy by huddling on the ground to minimize heat loss [9].

The selection of a common sleeping site relies on a coordinated collective movement, which often follows a structured leader–follower dynamic [10]. In this process, one individual typically initiates a new direction (e.g., toward a sleeping site), and others decide whether to follow, thereby participating in a collective choice [10]. Leadership is influenced by multiple factors including sex, age and social rank [10,11,12]. These influences are evident in collective decisions over sleeping sites across species. For instance, high-ranking individuals lead groups to sleeping sites in Taihangshan macaques (*Macaca mulatta tcheliensis*) [13], while in chimpanzees (*Pan troglodytes*) inhabiting high-risk anthropogenic areas, males with high social centrality indirectly shape spatial use [14]. Similarly, environmental and social factors shape sleeping-site selection; in meerkat groups (*Suricata suricatta*), they show a preference for central sleeping sites during pup-rearing and increase the frequency of switching sites when resources are scarce, with the dominant female exerting a particularly strong influence on these decisions [15].

Despite extensive research on the environmental characteristics of primate nocturnal sites and variations in sleep trees [4,16,17,18], a systematic investigation of the intrinsic decision-making processes driving their selection remains lacking [19]. Tibetan macaques (*Macaca thibetana*) represent an ideal model for filling this gap, prompting our study of their sleeping-site selection and behavioral decisions. First, the species follows a matrilineal social structure, wherein females remain in their natal group for life, accumulating extensive knowledge of sleeping sites, including their locations and associated safety. Second, Tibetan macaques display pronounced seasonal rhythms, characterized by distinct mating and non-mating seasons [20]. Females undergo significant seasonal shifts in physiological priorities, such as reproductive security, which can directly influence decision-making. To meet thermoregulatory demands in colder conditions, the monkeys exhibit a seasonal shift in their choice of sleeping site, transitioning from arboreal to terrestrial sites and utilizing group huddling to minimize heat loss [21]. While previous work from our research group has identified factors and processes related to visual and vocal communication during Tibetan macaque group movements [22,23], the decision-making mechanisms governing sleeping-site selection remain unexplored. This seasonal shift in sleeping-site type inherently alters group coordination demands, offering a valuable system for studying how social and ecological factors interact to shape decision-making across seasons.

In the present study, we investigated how seasonal variation (mating versus non-mating seasons) and sleeping-site type—as two critical contextual dimensions—jointly shape the decision-making process in one group of free-ranging Tibetan macaques, with specific emphasis on the roles of sex, age, dominance rank, and social centrality. We propose the following predictions: (1) Sleeping-site selection is influenced by varying environmental conditions, exhibiting seasonal shifts, with a preference for arboreal sites during warmer periods of both the mating and non-mating seasons, and terrestrial sites during colder periods. (2) Females serve as primary decision-makers, participating earlier in collective movements and attracting more followers. (3) The collective decision-making process for sleeping-site selection is driven by the interaction between seasons (with special focus on the mating season) and sleeping-site types (arboreal vs. terrestrial), and the influence patterns of socio-biological factors (including social centrality, individual rank, age, and sex) on the decision-making process vary significantly.

## 2. Materials and Methods

### 2.1. Subjects and Study Sites

The research was carried out between July 2024 and April 2025 in the Huangshan National Nature Reserve, Anhui Province, China. The study area is located in Huangshan, southern Anhui Province, eastern China (E 118°01′–118°17′, N 30°01′–30°18′), covering an area of approximately 160.6 km^2^ [24]. The study was conducted at Huangshan Wild Monkey Valley (30°29′ N, 118°10′ E), with the home range of Group YA1 being about 6 km^2^ [24]. This region is characterized by a subtropical monsoon climate, with complex mountainous topography. The dominant vegetation types are subtropical evergreen broad-leaved forests and deciduous mixed forests, which provide abundant food resources for Tibetan macaques. Potential predators in the study site include the clouded leopard (*Neofelis nebulosa*) and the dhole (*Cuon alpinus*) [20]. The study group uses arboreal sleeping sites dominated by *Cyclobalanopsis glauca* and *Pinus massoniana* during warm months, and shifts to terrestrial sleeping sites on flat, protruding cliff sections at elevations of 600–700 m as temperatures decrease [20,25].

The study area is inhabited by a total of 9 wild Tibetan macaque (*Macaca thibetana*) groups, namely YAI, YAII, TBSII, JLP, TLG, FRL, SG, HX and THII. Excluding Group YAI, the remaining eight groups have a total of 275–345 individuals. Among them, Groups YAII, TLG and FRL are distributed in the core tourist area of Huangshan Wild Monkey Valley (30°29′ N, 118°10′ E), adjacent to the core habitat of Group YAI, with potential home range overlap with the latter [24].

Data were collected on the Yulinkeng 1 (YA1) group, a well-habituated group monitored continuously since 1986. All adult individuals are individually recognizable based on unique physical characteristics, including scars, fur color, and facial morphology [20]. The study period was divided into two seasonal phases [20]: the mating season (July 2024–January 2025) and the non-mating season (February–April 2025). Based on local meteorological records, months with a mean monthly temperature above 15 °C (July–September 2024 and April 2025) were defined as warm months, and the remaining months (October 2024–March 2025) as cool months.

The YA1 group underwent slight group composition changes during the study. At the early stage of the mating season (July 2024) during the study period, the group had a total of 58 individuals with a female-to-male sex ratio of 22:36, including 10 adult males (aged ≥7 years), 11 adult females (aged ≥5 years), 11 subadults (aged 4–6 years), 9 juvenile females (aged 0–3 years) and 17 juvenile males (aged 0–3 years). At the late stage of the non-mating season (April 2025), the total number of individuals in the group decreased to 49, with a female-to-male sex ratio of 20:29. The group composition at this stage was as follows: 9 adult males, 12 adult females, 9 subadults, 8 juvenile females and 11 juvenile males. Our final research sample comprised 24 adult individuals (mentioned above) from this group (Table 1). In this study, the total effective observation time of focal individuals was 110.5 h, with a mean of 4.6 h per individual.

During the study period, we provided the Tibetan macaques with 3–4 kg of artificially supplied corn at 09:00, 11:00, 14:00 and 17:00 each day, and the corn was widely distributed in highly visible positions to prevent monopolization by single or a small number of high-ranking individuals.

### 2.2. Data Collection

#### 2.2.1. Behavioral Observation and Definition

All behavioral data for this study were collected by a single observer. Daily observation sessions were conducted from 8:30 to 11:30 and from 14:00 to 18:30, totaling 7.5 h per day, and observations were only suspended under extreme weather conditions (e.g., heavy rain, dense fog) that severely hindered behavioral recording and individual identification. In addition, there were days when the monkey group did not appear in the observation area due to the natural expansion of their foraging range, and no valid collective movement data were obtained on those days. For evening observations of collective movement from the provisioning site to the sleeping location, all-occurrence recording was used [27], with the entire process filmed from a video camera (Sony, China, Beijing). Data recorded the identities of movement initiators and followers, the timing of departures, and the sequence in which followers joined the procession. Detailed operational definitions for these behaviors are provided in Table 2 [22,28]. To determine the order and position at which individuals joined collective movements, we established a transect with distance markers along the primary path from the provisioning site to the forest edge. Data on collective movements toward sleeping sites were recorded only when more than 15 adults were observed, representing at least two thirds of the total adult population. The two-thirds group membership threshold was determined based on the relevant literature and empirical evidence in primate collective behavior research [29], ensuring that recorded events reliably represent the whole group rather than small subgroups.

Aggressive–submissive bouts were recorded using an ad libitum sampling method. Aggressive behaviors included staring, ground-slapping, chasing, scratching, or biting, while submissive behaviors were characterized by teeth-baring, avoidance, fleeing, or screaming [30]. An audio recording device (model News my V03) was used to observe the behaviors of the 24 focal individuals in this study. During each 10 min focal animal sampling period [27], researchers verbally recorded the social behaviors of the focal individuals in real time via the audio recording device, including the identities of all conspecific individuals within a 1 m proximity to the focal individuals. All audio records were subsequently transcribed verbatim and systematically entered into Excel spreadsheets for data collation.

#### 2.2.2. Types of Sleeping Sites

In this study, a sleeping site was defined as a specific location where the Tibetan macaque group ceases activity at dusk and rests until the following morning. According to established classifications [31], the sleeping sites of Tibetan macaques were primarily categorized into two types: cliff platforms and clusters of trees. Consequently, sites primarily utilized as sleeping areas on cliff platforms were classified as “terrestrial sleeping sites,” while those predominantly used within clusters of trees were designated as “arboreal sleeping sites”. Furthermore, research observations indicate that conspicuous fecal deposits are typically present at sleeping sites frequently utilized by Tibetan macaques, serving as auxiliary indicators for identifying habitual sleeping locations. No detailed habitat characteristics (e.g., canopy structure, cliff exposure, etc.) of either arboreal or terrestrial sleeping sites were recorded in this study.

### 2.3. Rank, Social Centrality and Age Classes

Individual dominance ranks for during both mating and non-mating seasons were calculated using David’s score (DS), based on dyadic agonistic interactions, where a higher DS value indicates a higher position in the social hierarchy [26].

In social network analysis, the Dyadic Association Index (DAI) between two individuals, A and B, is as follows:

DAI = Dab/(Da + Db − Dab)

where Dab represents the total time A and B spent within 1 m of each other, and Da and Db are the total focal sampling durations for A and B, respectively. This method is rooted in the foundational primatological research of Nishida [32], who first demonstrated the utility of spatial co-occurrence metrics for measuring social familiarity and bond strength in wild chimpanzee groups and established spatial proximity as a core indicator of primate social structure—a principle that underpins our fine-scale social association measurements for analyzing collective movement decision-making. Eigenvector centrality coefficients for during both mating and non-mating seasons were subsequently derived from the DAI matrix using Gephi 0.10.1. A higher eigenvector centrality coefficient indicates the individual had closer connection with others in the group [33] (Table 1).

Age classification was performed in accordance with Li [20], categorizing individuals into distinct age classes for subsequent analysis using the following numerical codes: “1” for adult (males: 7–10 years; females: 5–10 years), “2” for middle-aged (10–15 years), and “3” for old (≥15 years).

### 2.4. Joining Order

During collective movement toward the sleeping site, each follower was assigned a joining order score: The initiator was assigned position 0, and each subsequent joiner received position *j*, corresponding to the total number of individuals already moving when it joined [34]. Thus, a higher value of *j* indicates a later joining order in the collective movement.

### 2.5. Statistical Analysis

We performed conditional logistic regression using the survival package [35,36] in R version 4.3.3 (R Core Team 2023). The dependent variable was “role” (initiator = 1, follower = 0), with individual characteristics (sex, age, dominance rank, and eigenvector centrality) included as predictors. Analyses were stratified by sleeping-site type (arboreal/terrestrial), with “event ID” specified as the strata variable (survival package). Separate models were fitted for collective movement data during mating and non-mating seasons. The effects of predictors were evaluated by examining *p*-values and coefficients from the conditional logistic regression, with a *p*-value < 0.05 considered statistically significant.

We constructed generalized linear mixed models (GLMMs) and generalized linear models (GLMs) using the lme4 package in R to examine the effects of sex, age, DS, and social centrality on collective movement. Separate models were constructed for mating and non-mating seasons, and for arboreal and terrestrial sleeping-site types. For analyzing the number of followers and joining order, we used GLMMs with a Poisson distribution. In the model of the number of followers, initiator identity was included as the dependent variable; in the model of joining order, follower identity was treated as the dependent variable. Fixed factors included sex, age, DS, and social centrality. Prior to analysis, both social centrality and DS were standardized to a mean of 0 and a standard deviation of 1. All analyses were conducted in R version 4.3.3, with a significance threshold of α = 0.05.

## 3. Results

### 3.1. Seasonal Patterns in Sleeping Sites of Tibetan Macaques

**Figure 1:**
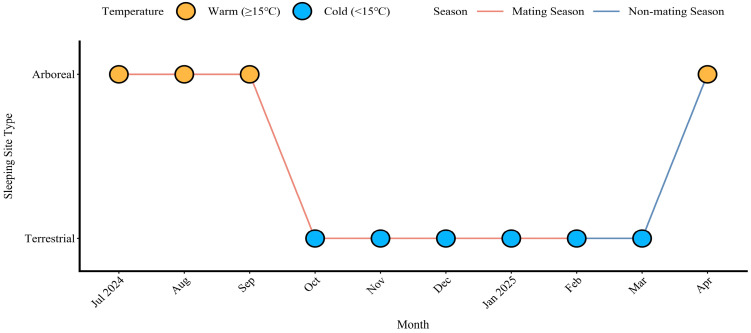
Seasonal variations in sleeping-site selection and temperature in Tibetan macaques.

A total of 186 successful collective movements to sleeping sites were observed in Tibetan macaques. Of these, 110 movements occurred during the mating season (July 2024–January 2025) and 76 movements during the non-mating season (February 2025–April 2025). Sleeping-site selection in both seasons showed a clear seasonal pattern correlated with environmental conditions. (Figure 1): During the mating season, the group selected arboreal sleeping sites in warmer months (July–September; 23 movements) and shifted to terrestrial sleeping sites in colder months (October–January; 87 movements). In the non-mating season, the group continued to use terrestrial sites during the colder months (February–March; 51 movements) and selected to arboreal sites in the warmer month of April (25 movements).

### 3.2. Influence of Social and Biological Factors on Decision-Making Across Seasons

**Figure 2:**
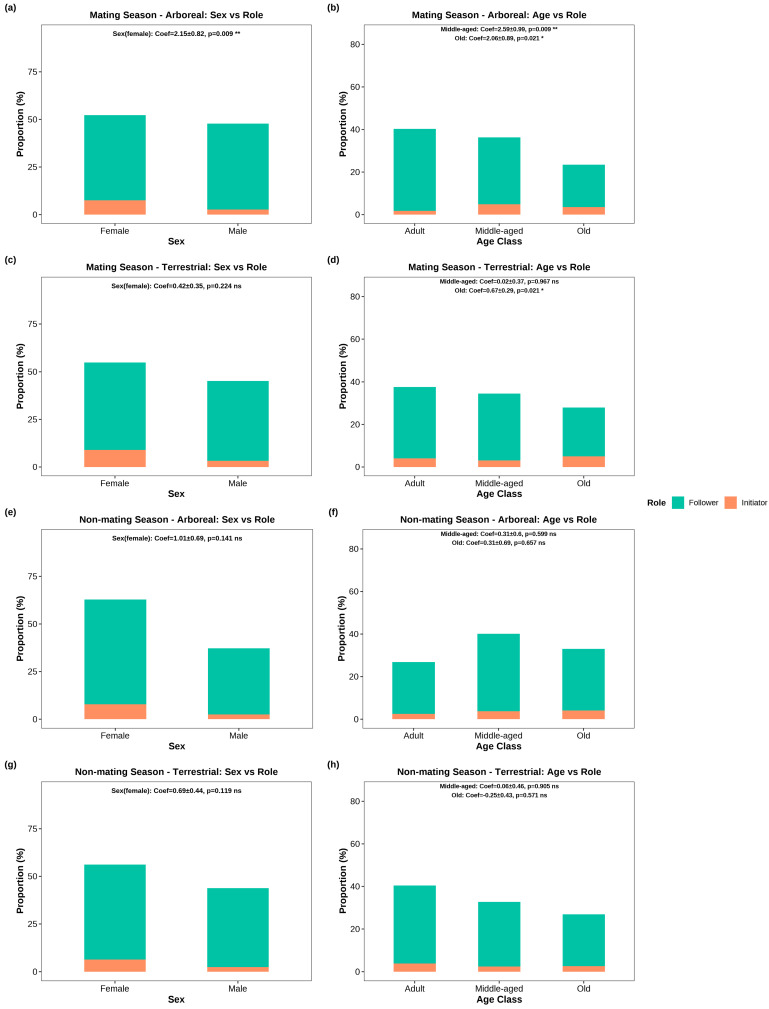
Proportions of initiators (orange) and followers (green) in Tibetan macaques during collective movements to sleeping sites, stratified by sex and age class under different seasons and sleeping-site types. (**a**) Mating season, arboreal: sex vs. role; (**b**) mating season, arboreal: age class vs. role; (**c**) mating season, terrestrial: sex vs. role; (**d**) mating season, terrestrial: age class vs. role; (**e**) non-mating season, arboreal: sex vs. role; (**f**) non-mating season, arboreal: age class vs. role; (**g**) non-mating season, terrestrial: sex vs. role; (**h**) non-mating season, terrestrial: age class vs. role. Statistical results (Coef, SE and *p*-values) are labeled at the top of each panel. * *p* < 0.05, ** *p* < 0.01. ns = not significant.

Regarding the roles of initiators and followers, females and middle-aged/older individuals were more likely to initiate movements to arboreal sleeping sites (female: coefficient = 2.15, *p* < 0.01; middle-aged: coefficient = 2.59, *p* < 0.01; older: coefficient = 2.06, *p* < 0.05; Figure 2a,b), while only older individuals led movements to terrestrial sleeping sites (coefficient = 0.67, *p* < 0.05; Figure 2d) during the mating season.

In contrast, none of the tested variables (sex, age, DS, social centrality) significantly predicted the initiator role for either arboreal or terrestrial sleeping sites (all *p* > 0.05; Figure 2e–h, Appendix A) during the non-mating season.

The results of the conditional logistic regression model for initiators/followers are presented in Appendix A.

**Figure 3:**
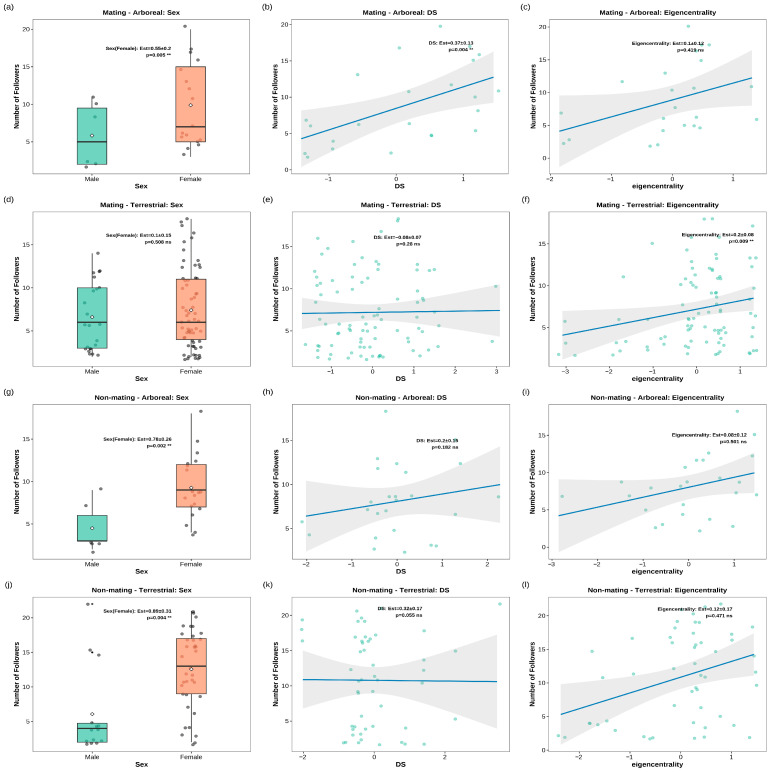
Relationships between the number of followers and sex, social rank (DS), and social centrality (eigencentrality) under different seasonal and sleeping-site conditions. (**a**–**c**) Mating season, arboreal sites; (**d**–**f**) mating season, terrestrial sites; (**g**–**i**) non-mating season, arboreal sites; (**j**–**l**) non-mating season, terrestrial sites. Box plots show the distribution of follower numbers across sex groups, with jittered points representing individual observations and white diamond points indicating group means. Scatter plots with linear regression lines show the relationships between continuous variables (DS/eigencentrality) and follower numbers. Scattered points represent raw data, and gray shaded areas indicate 95% confidence intervals. Statistical results (Estimate, SE, and *p*-values) are labeled at the top of each panel ** *p* < 0.01, ns = not significant.

For the number of followers, sex (GLM, Z = 2.78, *p* < 0.01) and dominance rank (DS; GLM, Z = 2.88, *p* < 0.01) had significant effects, with females and higher DS individuals attracting more followers at arboreal sleeping sites during the mating season (Figure 3a,b). Conversely, only social centrality (eigencentrality) emerged as a significant predictor, with individuals exhibiting higher social centrality attracting more followers at terrestrial sleeping sites during the same season (GLMM, Z = 2.61, *p* < 0.01; Figure 3f).

During the non-mating season, females attracted significantly more followers than males in both arboreal (GLM, Z = 3.04, *p* < 0.01; Figure 3g) and terrestrial (GLMM, Z = 2.86, *p* < 0.01; Figure 3j) sleeping-site contexts. No significant effects of dominance rank, social centrality (all *p* > 0.05; Figure 3h,i,k,l) and age (*p* > 0.05) on follower count were observed during the non-mating season.

The results of the generalized linear mixed model and generalized linear model for the number of followers are presented in Appendix B.

For joining order, females joined collective movements significantly earlier than males across both seasons and sleeping-site types (mating season—arboreal: *Z* = −2.06, *p* < 0.05; mating season—terrestrial: *Z* = −4.23, *p* < 0.001; non-mating season—arboreal: *Z* = −2.30, *p* < 0.05; non-mating season—terrestrial: *Z* = −1.97, *p* < 0.05) (Table 3). In the mating season—terrestrial model, both high DS (GLMM: *Z* = −2.83, *p* < 0.01) and older individuals (GLMM: *Z* = −4.43, *p* < 0.001) joined movements earlier. In contrast, during the non-mating season for terrestrial sites, older individuals joined significantly later (GLMM: *Z* = 2.98, *p* < 0.01), whereas those with higher social centrality joined earlier (GLMM: *Z* = −3.64, *p* < 0.001) (Table 3).

## 4. Discussion

This study examined the collective movement of wild Tibetan macaques to different types of sleeping sites, systematically investigating the interacting effects of social, biological, and seasonal factors on the decision-making process of sleeping-site selection. Our results support our first prediction, indicating that, in response to changes in environmental conditions, thermoregulation may potentially influence the sleeping-site selection of Tibetan macaques: During the mating season, arboreal sleeping sites were preferred in warmer conditions, while terrestrial sleeping sites were chosen in colder conditions; when temperatures increased outside the mating season, the group selected arboreal sleeping sites. Our second prediction was partially supported: Females played a central role in decision-making, initiating movement more often and attracting more followers in movements to arboreal sleeping sites during the mating season, though no significant advantage was observed in movements to terrestrial sleeping sites. Notably, females consistently joined movements at earlier stages across all seasons and sleeping-site types. The results also partially supported our third prediction, revealing that decision-making strategies and leadership allocation are shaped by seasonal variation and sleeping-site type.

### 4.1. Seasonal Patterns in Sleeping-Site Selection of Tibetan Macaques

Our study of 186 collective movements of Tibetan macaques toward sleeping sites reveals a distinct seasonal pattern in sleeping-site selection: Arboreal sites are preferred during the warmer months (July to September and the following April), while terrestrial sites are chosen as temperatures decline from October to March (Figure 1). Unlike most primates that exclusively sleep in trees [6], the study group regularly adopted terrestrial sleeping in cold conditions. This behavioral shift in colder periods, potentially linked to thermoregulatory benefits, aligns with observations in Japanese macaques (*Macaca fuscata*), which also exhibit huddling behavior for sleeping in cold conditions [9]. In low-temperature environments, group huddling can reduce heat loss and help maintain stable body temperatures [21,37,38]. Although the current study area experiences low predation risk, primates’ innate anti-predator fear tendency could still partially influence their sleeping-site selection. In chimpanzees (*Pan troglodytes*), for instance, relaxed predation pressure is associated with increased terrestrial sleeping [39]. This aligns with findings in Azara’s owl monkeys (*Aotus azarae azarae*), where sleeping-site selection reflects a trade-off between thermoregulation and predator avoidance [7]. In warmer periods, arboreal sleeping sites likely offer dual advantages: enhanced airflow for heat dissipation and reduced injury risk from venomous snakes [25]. Our study shows that Tibetan macaques move to terrestrial sleeping sites as temperatures drop. This is an important strategy for adapting to seasonal environmental changes. Sleeping-site selection shifts between mating and non-mating seasons, influenced by environmental conditions. This shift is likely influenced by multiple ecological factors. These include thermoregulatory needs, predator avoidance, and potential social influences.

### 4.2. Females’ Core Role in Tibetan Macaques’ Sleeping-Site Selection Decision-Making

Our results demonstrate that females played a central role in sleeping-site decision-making. They consistently exhibited a significant advantage by joining collective movements earlier than males across all seasons and sleeping-site types (Table 3). This behavior aligns with the matrilineal social structure of Tibetan macaques: Females remain in their natal group throughout their lives, except during group fission period, whereas males disperse upon reaching sexual maturity [20]. Consequently, females accumulate extensive knowledge about surroundings, enabling them to play a stabilizing role in sleeping-site selection decisions. These observations are consistent with Sueur’s [40] proposal that individuals with more pressing core needs (e.g., survival and reproduction) tend to exert a greater influence on group movement decisions. Similar phenomena have been observed in other species, such as yellow baboons (*Papio cynocephalus*) and plains zebras (*Equus burchellii*), where lactating females frequently occupy leading positions in movement sequences [41,42]. This behavior enables them to detect environmental risks earlier, guide the group toward resources, and indirectly shape the overall movement trajectory. In Tibetan macaques, females’ rapid recognition and earlier participation in collective movements toward sleeping sites reduce decision-making time within the group, enhancing movement synchrony and overall safety.

Furthermore, females consistently attracted more followers, except for during the mating season at terrestrial sleeping sites (Figure 3a,d,g,i). This is supported by previous research by Chen et al. [23], which found that female Tibetan macaques are more proficient in using vocal signals to coordinate group movement. The efficient transmission of information through vocal signals facilitates rapid synchronization of group intentions, reduces decision-making time, enhances movement coordination and safety, and ensures the cohesiveness of collective movements. Ultimately, this result underscores the central role of females in maintaining group cohesion. A similar pattern has been observed in other matrilineal species, such as bonobos (*Pan paniscus*) and Verreaux’s sifakas (*Propithecus verreauxi*) where females also play a central role in coordinating group movement [43,44]. The findings of this study suggest that the central role of females in sleeping-site decision-making may result from the combined effects of their ecological knowledge advantage (inherent in the matrilineal social structure), their stable behavioral influence across seasons, and their efficient group coordination mechanisms.

### 4.3. Mating Seasonal Differences in Sleeping-Site Selection Decisions of Tibetan Macaques: Arboreal vs. Terrestrial

Our findings revealed that decision-making processes in Tibetan macaques varied substantially by sleeping-site type during the mating season, reflecting distinct ecological and social trade-offs associated with each site type. Specifically, females and middle-aged/older individuals were more likely to initiate movement (Figure 2a,b), while higher-ranking (DS) individuals attracted more followers (Figure 3b), in collective movements to arboreal sleeping sites. In contrast, older individuals emerged as the primary initiators (Figure 2d), and individuals with higher social centrality attracted more followers (Figure 3f), during movements to terrestrial sleeping sites. This divergence in leadership allocation suggests that different individual attributes become advantageous depending on the specific demands imposed by each sleeping environment.

Most females, carrying highly dependent 3–4-month-old infants, were more inclined to initiate movement toward arboreal sleeping sites. This behavior was driven by their strong motivation to protect their offspring amidst intense male–male competition during the mating season, coupled with their familiarity with the environment. Arboreal sleeping sites, with their tall canopies and low ground exposure, allow females to sleep with their infants on the same branch [25]. This likely provides females with enhanced protection against conspecific male aggression and venomous snake infestations, making such sites a safer option for females prioritizing infant survival under warm conditions. This pattern parallels findings in ring-tailed lemurs *(Lemur catta*) and red-fronted lemur (*Eulemur rufifrons*), where females also play a leading role in group decision-making [45,46]. Meanwhile, middle-aged and older individuals contributed their experience in predator vigilance, supplementing safety assessments to successfully initiate collective movements, as similarly observed in European bison (*Bison bonasus*) [1] and bonobos (*Pan paniscus*) [43]. Additionally, intensified social competition during the mating season resulted in high-ranking individuals attracting more followers, likely due to their ability to provide stronger social alliance protection [47].

In contrast, older individuals were significantly more likely to become initiators and joined movements earlier (Figure 2d; Table 3), while individuals with higher social centrality attracted more followers during collective movements to terrestrial sleeping sites in the mating season (Figure 3f). Although females, burdened with infant care, continued to join early to ensure offspring safety, leadership was allocated to older individuals who were more sensitive to the cold. This shift in leadership reflects the distinct ecological pressures of cliff-based terrestrial sleeping sites: The rugged, confined terrain increases movement complexity, while post-sunset temperature drops accelerate body heat loss, creating urgent physiological demands for older individuals with higher energy expenditure [48]. Research by Languille et al. [48] indicates that short-day and cold conditions increase energy expenditure in older individuals, and older mouse lemurs (*Microcebus murinus*) show a preference for warmer nest sites [49]. The motivation for older individuals to initiate movement is closely linked to physiological demands under low temperatures. After sunset, ambient temperature drops accelerate body heat loss [50]. In response, behavioral thermoregulation (e.g., through huddling and shared shelter use) serves as a key adaptive strategy [50,51]. The study indicates that older Tibetan macaques, due to higher energy expenditure and cold sensitivity, were more motivated to initiate movement toward cliff-based terrestrial sleeping sites offering thermal shelter and wind protection.

Furthermore, individuals with higher eigenvector centrality attracted more followers during collective movements to terrestrial sleeping sites in the mating season (Figure 3f). Eigenvector centrality coefficient measures the closeness of an individual’s connections with other members within the group [52]. In the context of collective movements to cliff-based terrestrial sleeping sites—which often require navigating complex and hazardous paths—the group’s reliance on such socially central individuals may stem from multiple mechanisms. First, individuals with high centrality may serve as reliable information hubs, facilitating the transmission of spatial and environmental knowledge through both vocal and visual signals [22,23]. Second, their extensive social ties may generate greater trust or social attraction, making other group members more willing to follow their lead, particularly in situations requiring rapid decision-making. Third, following socially central individuals promoted larger huddles [21], enhancing thermal benefits for the whole group. Thus, the observed attraction of followers to high-centrality individuals reflects not only potential information advantages but also the broader social cohesion and coordination capacity embedded in their network position. This may be attributed to the complex and hazardous cliff paths in this environment, which heighten the group’s dependence on individuals capable of effective information transfer and guidance, individuals whose social connectivity and functional roles in the group’s social network structure are critical to maintaining collective movement cohesion and hierarchical coordination [53]. However, we acknowledge that eigenvector centrality represents a composite social metric shaped by multiple interacting dimensions, including spatial proximity, affiliative interactions, grooming relationships, and dominance dynamics. Future work employing more nuanced, component-specific analyses of social centrality will help clarify the distinct pathways through which different aspects of social connectedness contribute to leadership emergence and follower attraction during collective sleeping-site selection.

This context-dependent variation in leadership reveals the complexity of ecological and social trade-offs in the sleeping-site decision-making process of Tibetan macaques. These macaques flexibly allocate decision-making roles according to the match between individual traits and the specific ecological requirements of each sleeping site, thereby balancing multiple demands including infant safety, cold adaptation, potential predator avoidance, and terrain complexity. Clarifying the regulatory effect of sleeping-site type on leadership allocation can further highlight the ecological and social trade-offs that shape the collective decision-making behavior of this species.

### 4.4. Non-Mating Seasonal Leadership in Tibetan Macaques: Loss of Socio-Biological Predictors for Roosting Site Movement Initiation

This study found that, during the non-mating season, female macaques initiated movements toward sleeping sites at a higher proportion than males in both arboreal and terrestrial contexts (Figure 2e,g). However, conditional logistic regression models revealed that none of the tested socio-biological factors (sex, age, dominance rank, or social centrality), significantly predicted initiator identity (Appendix A; Figure 2f,h). This result is intriguing given the stable matrilineal structure of Tibetan macaques [20], in which females remain in their natal groups and accumulate long-term ecological knowledge across seasons. If social structure remains stable, why do sex and other socio-biological factors lose predictive power for initiator identity during the non-mating season?

We propose that this seasonal shift primarily reflects changes in social tension and reproductive priorities rather than changes in social structure itself. During the mating season, heightened male–male competition [47] and the presence of vulnerable infants collectively increase the demand for experience-based leadership. Females carrying infants may be more motivated to initiate movements toward safer locations, while middle-aged and older individuals may lead to fulfill thermoregulatory needs [49,50]. This motivational divergence, driven by reproductive pressure and physiological demands, enables specific social traits to serve as effective predictors of leadership during the mating season. In contrast, during the non-mating season, social tension relaxes and dependent infants are absent, reducing selective pressure for specific individuals to assume initiator roles. As demonstrated in red-fronted lemurs (*Eulemur rufifrons*), even in species where females generally lead group movements year-round, the pool of initiators expands during the non-breeding weaning period, and the demand for specific leaders diminishes significantly [46]. In this context, initiation events become more distributed among group members, obscuring the predictive role of social traits.

This study demonstrates that both ecological conditions (e.g., temperature variation) and social context (e.g., social tension, reproductive priorities, infant dependency) undergo seasonal changes that are critical for understanding seasonal variation in collective movement patterns. The contrasting patterns between mating and non-mating seasons highlight that leadership is not a fixed attribute of certain individuals but rather a context-dependent phenomenon shaped by the distribution of motivational urgency within the group.

## 5. Conclusions

In summary, the decision-making process regarding sleeping-site selection in Tibetan macaques represents a dynamic outcome of the interaction between their matrilineal social structure and seasonal ecological pressures. The main finding of this study reveals a clear division of roles and dynamic decision-making rules: Females play a central role in selecting sleeping sites, while leadership shifts among specific individuals according to ecological demands, forming a context-dependent decision-making mechanism. This provides key empirical evidence for the seasonal adaptation dimension of collective decision-making in primates and establishes a foundation for further investigation into inter-group decision-making differences. Nevertheless, the present study mainly focused on the socio-biological and seasonal factors shaping sleeping-site selection. Future studies should systematically quantify and document the habitat characteristics of both arboreal and terrestrial sleeping sites (e.g., canopy structure, cliff exposure, etc.), to further clarify how predation risk and habitat features interact with social factors to influence sleeping-site decisions in Tibetan macaques. Future research can further verify the role of thermoregulatory mechanisms in the sleeping-site selection of Tibetan macaques, and rigorously test these mechanisms by quantifying microclimatic conditions, assessing the relative safety of distinct sleeping sites, analyzing the effects of interannual climatic variations (e.g., extreme low temperatures and prolonged rainy seasons), and examining the influence of sleeping postures on thermoregulation. Such research will not only facilitate an in-depth analysis of the multi-layered driving factors underlying the group movement decisions of Tibetan macaques, but also validate the long-term stability of relevant findings, while exploring how the interplay between climatic and social factors shapes the dynamic processes of their collective decision-making. In addition, future studies should incorporate data on the spatial locations of sleeping sites and their distances from feeding sites to systematically investigate their influences on sleeping-site selection decisions in Tibetan macaques.

## Figures and Tables

**Table 1:** Characteristics of the focal subjects from Group YA1.

Individuals	Sex	Age Class ^#^	David’s Score *		Eigenvector Centrality	
			Mating Season	Non-Mating Season	Mating Season	Non-Mating Season
WM	Male	Old	64.60	14.77	0.58	0.92
BM	Male	Old	−57.82	−9.51	0.93	0.65
YL	Male	Middle-aged	141.75	58.78	1.00	0.88
YXK	Male	Middle-aged	136.65	38.28	0.96	0.89
TQ	Male	Middle-aged	69.75	12.83	0.89	0.64
NM	Male	Middle-aged	52.61	1.80	0.82	0.79
TQS	Male	Middle-aged	25.25	/	0.92	/
YXM	Male	Adult	−7.59	−7.04	0.96	0.27
LB	Male	Adult	−11.14	−15.91	0.75	0.36
YQT	Male	Adult	−39.75	−12.11	0.80	0.53
DZ	Male	Adult	−69.04	−11.21	0.85	0.45
YG	Male	Adult	/	−7.08	/	0.47
TXH	Female	Old	29.05	−3.75	0.67	0.93
TXX	Female	Old	8.75	−7.75	0.96	0.82
TH	Female	Old	−19.70	−11.92	0.59	0.72
THY	Female	Old	−63.86	−35.75	0.81	0.74
YCH	Female	Middle-aged	30.50	22.33	0.69	1.00
YCL	Female	Middle-aged	10.19	2.88	0.93	0.74
THX	Female	Middle-aged	−71.12	−11.58	0.82	0.55
TQL	Female	Adult	−9.00	−8.87	0.92	0.83
TQG	Female	Adult	−15.84	−6.47	0.94	0.80
TFH	Female	Adult	−49.50	−0.67	0.76	0.61
TQZ	Female	Adult	−53.50	−2.00	0.93	0.80
TQY	Female	Adult	−55.05	−0.25	0.69	0.49

^#^ Adult: males aged 7–10 years and females aged 5–10 years; middle-aged: male and female ≥ 10~15 years old; old: male and female ≥ 15 years old. * David’s score [26] was used to quantify dominance ranks, with a higher DS denoting a higher position in the social hierarchy.

**Table 2:** Behavioral definitions for collective movement in Tibetan macaques [22,28].

Catalog	Definition
Collective movement	The collective movement was considered complete once five minutes had passed since the last individual joined, provided that at least three individuals were participating.
Initiator	The initiator was defined as the first individual observed to travel more than 10 m within a 30s period.
Follower	A following behavior was defined as a focal individual moving > 5 m in the initiator’s departure direction, with an angular deviation of <45°, prior to the collective movement’s termination.

**Table 3:** Results of GLMM analyses examining the effects of social factors on joining sequence across seasons and sleeping-site types.

Model	Factor	Estimate	SE	*Z* Value	*p*-Value
Mating season—Arboreal	(Intercept)	2.06	0.14	14.88	<0.001 ***
	Sex (female)	−0.34	0.16	−2.06	<0.05 *
	Age2	−0.26	0.18	−1.422	0.16
	Age3	−0.29	0.15	−1.91	0.06
	DS	−0.03	0.09	−0.30	0.76
	Centrality	0.05	0.08	0.62	0.544
Mating season—Terrestrial	(Intercept)	1.78	0.04	39.57	<0.001 ***
	Sex (female)	−0.23	0.05	−4.23	<0.001 ***
	Age2	−0.10	0.06	−1.60	0.11
	Age3	−0.23	0.05	−4.43	<0.001 ***
	DS	−0.08	0.03	−2.83	<0.01 **
	Centrality	−0.01	0.03	−0.36	0.72
Non-mating season—Arboreal	(Intercept)	1.69	0.14	11.89	<0.001 ***
	Sex (female)	−0.30	0.13	−2.30	<0.05 *
	Age2	0.15	0.13	1.13	0.26
	Age3	0.22	0.15	1.50	0.13
	DS	−0.07	0.08	−0.86	0.39
	Centrality	−0.06	0.07	−0.95	0.34
Non-mating season—Terrestrial	(Intercept)	1.86	0.10	18.70	<0.001 ***
	Sex (female)	−0.20	0.10	−1.97	<0.05 *
	Age2	0.20	0.11	1.76	0.08
	Age3	0.36	0.12	2.98	<0.01 **
	DS	−0.03	0.07	−0.43	0.67
	Centrality	−0.23	0.06	−3.64	<0.001 ***

Significance codes: *** *p* < 0.001, ** *p* < 0.01, * *p* < 0.05.

## Data Availability

Data will be made available on request.

## Appendix A

**Table A1:** Results of conditional logistic regression analysis of the effects of social factors on initiator/follower roles across seasons and sleeping-site types.

Model	Factor	Coefficient	Exp (Coef.)	Std Err (Coef.)	*p*-Value
Mating season—Arboreal	Sex (female)	2.15	8.60	0.82	<0.01 **
	Age2	2.59	13.30	0.99	<0.01 **
	Age3	2.06	7.84	0.89	<0.05 *
	DS	0.37	1.45	0.39	0.34
	Centrality	0.20	1.22	0.40	0.63
Mating season—Terrestrial	Sex (female)	0.42	1.52	0.35	0.22
	Age2	0.02	1.02	0.37	0.977
	Age3	0.67	1.95	0.29	<0.05 *
	DS	−0.19	0.82	0.19	0.31
	Centrality	0.32	1.37	0.17	0.07
Non-mating season—Arboreal	Sex (female)	1.01	2.76	0.69	0.14
	Age2	0.31	1.37	0.60	0.60
	Age3	0.31	1.36	0.69	0.66
	DS	0.07	1.07	0.40	0.86
	Centrality	0.25	1.29	0.3	0.5
Non-mating season—Terrestrial	Sex (female)	0.69	2.00	0.44	0.12
	Age2	0.06	1.06	0.46	0.90
	Age3	−0.25	0.78	0.43	0.57
	DS	−0.23	0.79	0.30	0.44
	Centrality	0.48	1.62	0.27	0.07

Significance codes: ** *p* < 0.01, * *p* < 0.05.

## Appendix B

**Table A2:** Results of GLM and GLMM analyses examining the effects of social factors on the number of followers across seasons and sleeping-site types.

Model	Factor	Estimate	SE	*Z* Value	*p*-Value
Mating season—Arboreal	(Intercept)	1.68	0.27	6.25	<0.001 ***
	Sex (female)	0.55	0.20	2.78	<0.01 **
	Age2	−0.05	0.27	−0.17	0.86
	Age3	−0.06	0.25	−0.24	0.81
	DS	0.37	0.13	2.88	<0.01 **
	Centrality	0.10	0.12	0.81	0.42
Mating season—Terrestrial	(Intercept)	1.71	0.17	9.94	<0.001 ***
	Sex (female)	0.10	0.15	0.66	0.51
	Age2	0.31	0.20	1.53	0.13
	Age3	0.21	0.15	1.3	0.17
	DS	−0.08	0.07	−1.08	0.28
	Centrality	0.20	0.08	2.61	<0.01 **
Non-mating season—Arboreal	(Intercept)	1.59	0.31	5.09	<0.001 ***
	Sex (female)	0.78	0.26	3.04	<0.01 **
	Age2	−0.26	0.22	−1.17	0.24
	Age3	−0.18	0.26	−0.67	0.5
	DS	0.20	0.15	1.34	0.18
	Centrality	0.08	0.12	0.67	0.50
Non-mating season—Terrestrial	(Intercept)	1.78	0.29	6.15	<0.001 ***
	Sex (female)	0.89	0.31	2.86	<0.01 **
	Age2	−0.58	0.34	−1.70	0.09
	Age3	−0.04	0.23	−0.17	0.86
	DS	0.32	0.17	1.92	0.05
	Centrality	0.12	0.17	0.72	0.47

Significance codes: *** *p* < 0.001, ** *p* < 0.01.
